# Pin tract infection of operatively treated supracondylar fractures in children: long-term functional outcomes and anatomical study

**DOI:** 10.1007/s11832-015-0674-8

**Published:** 2015-08-09

**Authors:** Shital N. Parikh, Marios G. Lykissas, Mazen Roshdy, Ronald C. Mineo, Eric J. Wall

**Affiliations:** Division of Orthopaedic Surgery, Cincinnati Children’s Hospital Medical Center, 3333 Burnet Avenue MLC 2017, Cincinnati, OH 45229 USA

**Keywords:** Supracondylar fracture, Pin tract infection, Percutaneous pinning, Septic arthritis, Intracapsular

## Abstract

**Purpose:**

The purpose of our study was to determine the long-term functional outcomes of pin tract infection after percutaneous pinning of displaced supracondylar humeral fractures in children, and to evaluate the potential for intracapsular pin placement based on pin configuration in cadaveric elbows.

**Methods:**

We conducted a retrospective review of all patients requiring percutaneous pinning in a single institution over a 19-year period. The functional outcome assessment consisted of a telephone interview using the Disabilities of the Arm, Shoulder and Hand (DASH)] Outcome Measure and the Patient-Rated Elbow Evaluation (PREE) questionnaires. The risk of intracapsular pin placement was studied in cadaveric elbows for the three most common pin configurations: divergent lateral, parallel lateral, and medial and lateral crossed pins.

**Results:**

Of 490 children, 21 (4.3 %) developed pin tract infection. There were 15 (3.1 %) superficial and six (1.2 %) deep infections (osteomyelitis and septic arthritis). Both DASH and PREE scores were excellent at a mean of 18 years post-surgery. The risk of intracapsular pin placement using parallel lateral pins was found to be greater (*p* < 0.05) than either crossed or divergent lateral pinning configurations.

**Conclusions:**

Most infections after pinning of supracondylar humerus fractures are superficial and can be managed with pin removal, oral antibiotics, and local wound care. Septic arthritis and osteomyelitis are rare complications; when they do occur, they seem to be associated with parallel lateral pin configuration, though a causal relationship could not be established from the current study. Satisfactory long-term outcomes of these deep infections can be expected when treated aggressively with surgical debridement and intravenous antibiotics.

## Introduction

Supracondylar fractures of the distal humerus are the most common elbow fractures in children, accounting for about 60 % of all elbow fractures [1]. Closed reduction and percutaneous pinning has become the standard of care for displaced supracondylar fractures [2–4]. This approach has reduced the incidence of cubitus varus and limb-threatening ischemia [3, 5, 6]. However, percutaneous pin fixation has led to a unique set of complications, including pin tract infection, hardware failure (or pin migration), and iatrogenic nerve injuries [4, 6–10].

Pin tract infection is the most common complication associated with percutaneous pin fixation of fractures in children, ranging from 1 to 21 % [11]. Battle and Carmichael [12] reported 16 pin tract infections in a series of 202 fractures in children, a rate of 7.9 %. Sharma et al. [13] reported six pin tract infections after pinning of 105 upper extremity fractures in children. For percutaneous pinning of supracondylar humerus fractures, infection rates have varied from 0 to 8 % [4, 6, 14–16]. Although pin tract infection is a common complication, long-term functional outcomes and the risk factors for developing septic arthritis or osteomyelitis have not been investigated. The purpose of our study was to determine the long-term functional outcomes of pin tract infection after percutaneous pinning of displaced supracondylar humeral fractures in children. A second objective was to evaluate the potential for intracapsular pin placement with the three common pin configurations used in clinical practice, using cadaveric elbows.

## Materials and methods

### Clinical study

After institutional review board approval was obtained, a computerized medical record search was performed to identify all supracondylar humerus fractures that underwent closed reduction and percutaneous pinning between January 1983 and April 2002. Open fractures, fractures that required open reduction, and condyle and epicondyle fractures were excluded.

Patients who developed pin tract infection after percutaneous pinning of supracondylar humerus fractures were identified after a thorough review of the medical records. Medical records were also reviewed for patient demographic information, time delay from presentation to surgery, preoperative antibiotic administration, number and configuration of pins, and fracture classification. Their clinical presentation was recorded, as well as the type of infection and its subsequent treatment. Radiographs were reviewed to identify the type of fracture and pin configuration.

The functional outcome assessment consisted of a telephone interview conducted with the patient using the Disabilities of the Arm, Shoulder and Hand (DASH) Outcome Measure [17] and the Patient-Rated Elbow Evaluation (PREE) [18]. The DASH is a standardized questionnaire comprising 30 items, all of which are scored using a five-point scale (1–5). The sum of the response values is used to calculate an initial score, which is then transformed to obtain the DASH score. DASH scores range from 0 (best function) to 100 (worst function). The PREE consists of two sections, evaluating pain and function. It contains 20 questions scored on a ten-point scale (0–10). The total score ranges from 0 to 200, with higher scores indicating worse functioning. In addition to the two questionnaires, we inquired about cosmesis and scarring, range of motion and stiffness, pain or analgesic use, and activity limitations.

### Cadaveric study

**Fig. 1:**
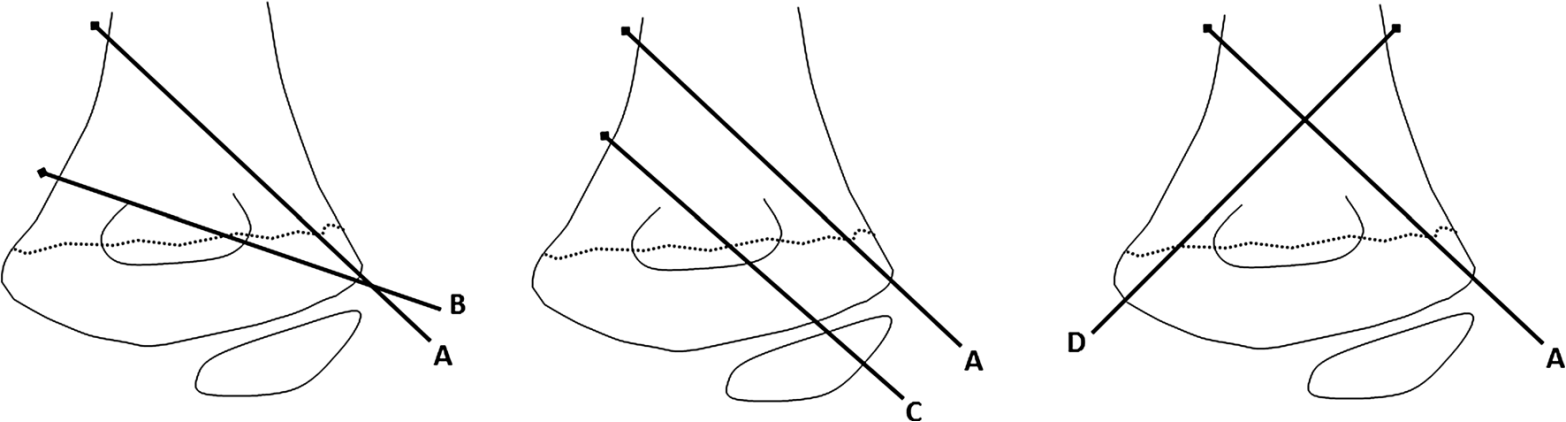
Schematic diagram showing the three different pin configurations: divergent lateral (pins *A* and *B*), parallel lateral (pins *A* and *C*) and crossed pins (*pins* *A* and *D*)

In order to evaluate the potential for capsular penetration and intracapsular pin placement of the three common pin configurations used in clinical practice, six upper limbs from three fresh adult cadavers were used. There was no known history of traumatic injury or joint disease of the elbow. Three left and three right elbows were evaluated. Four pins were placed by one of the authors (SNP) in each elbow to simulate the three most common pin configurations: divergent lateral, parallel lateral, and crossed-pin configuration. Pin A was placed from the lateral side of the elbow to simulate the most lateral (radial) pin in all three configurations. Pin B was inserted from the lateral side of the elbow to simulate divergent pin configuration. Pin C represented parallel pin configuration, and pin D was the medial pin of the crossed-pin configuration (Fig. 1).

**Fig. 2:**
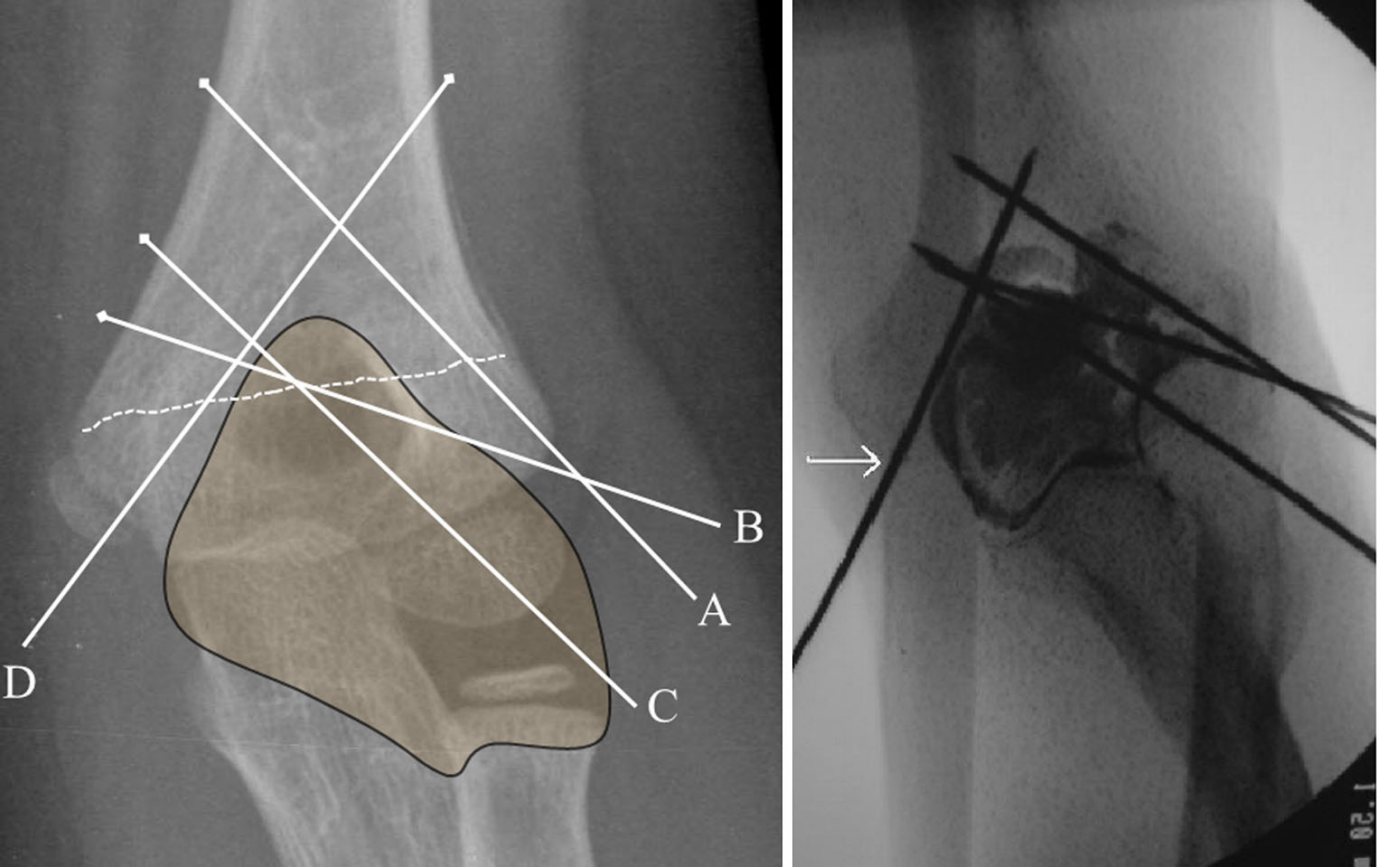
Anteroposterior radiograph demonstrating the relationship of the pins to the capsule. The arthrogram shows that the medial pin (*white arrow*) is extracapsular. Laterally,* pins A * and* B* appear to be extracapsular, and* pin C* appears to be intracapsular

**Fig. 3:**
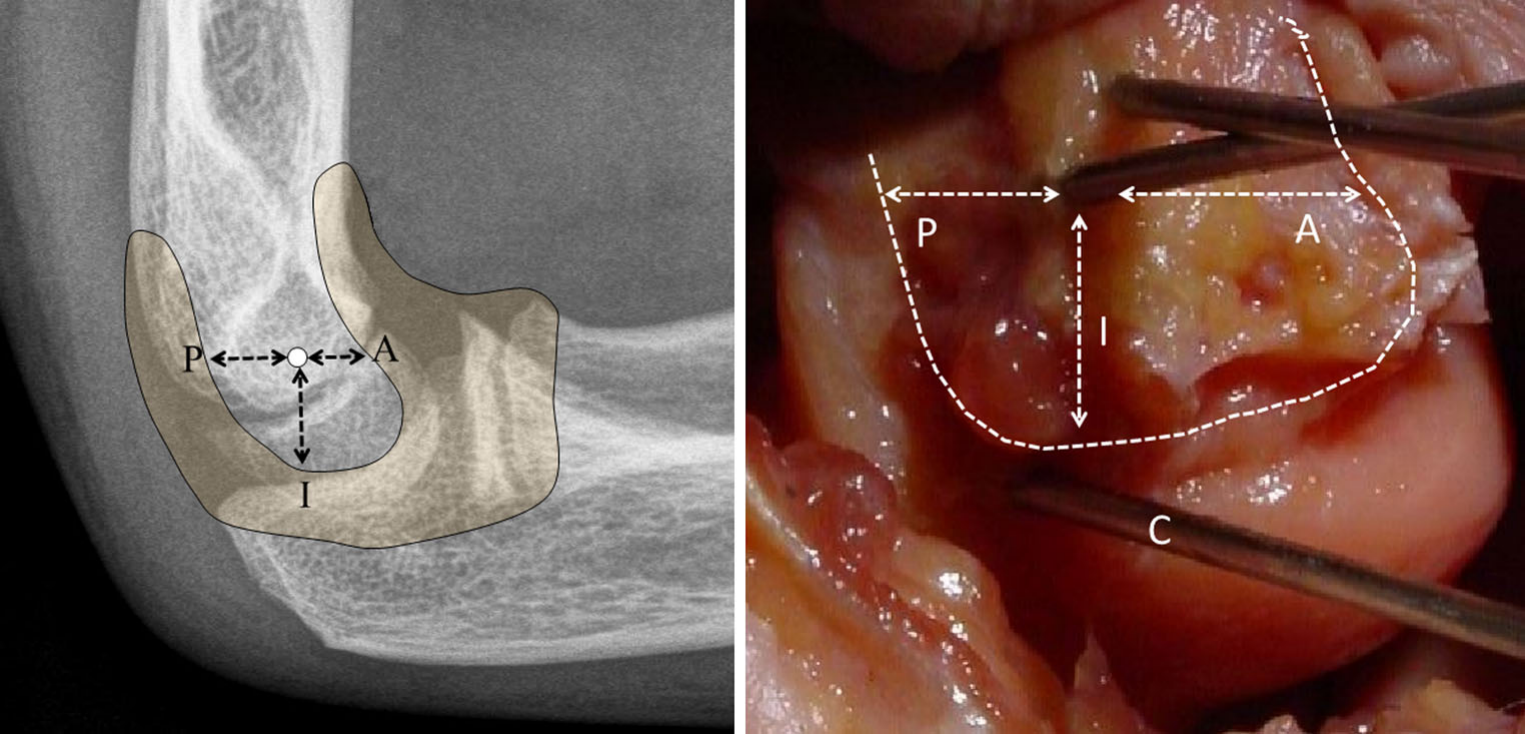
A lateral radiograph and dissection demonstrates capsular reflection and the anterior (*A*), posterior (*P*) and inferior (*I*) distance between the pin and the capsule. *Pin C* is intracapsular, as seen in the dissected elbow

Once all four pins were placed, an arthrogram was performed under fluoroscopic image guidance by injecting 5 ml of Conray contrast media (Mallinckrodt Pharmaceuticals, St. Louis, MO, USA) into the elbow joint (Fig. 2). This was followed by dissection of both the lateral and medial aspect of the elbow to determine the distance of each pin from the elbow joint capsule. Each lateral pin–capsule distance was measured from the anterior, inferior, and posterior margins of the capsule (Fig. 3). The medial pin-to-capsule distance was measured from the medial margin of the capsule. Statistical comparison of different groups was performed using two-tailed Wilcoxon signed-rank tests. This non-parametric test was selected due to the small sample size and non-normal distribution of data, and accordingly, medians and interquartile ranges (IQR) are reported instead of means and standard deviations. In all instances, *p* < 0.05 was regarded as statistically significant.

## Results

### Clinical study

Over a 19-year period (1983–2002), we identified 21 of 490 children (4.3 %) who developed pin tract infection after closed reduction and percutaneous pin fixation for displaced supracondylar humerus fracture at our institution. The mean age of these 21 children at the time of the injury was 5.0 ± 2.7 years (range 1–11 years). Thirteen were male and eight were female. Eleven cases involved the left side and ten the right. According to Wilkins' modification of the Gartland classification, 8 were type II and 13 were type III fractures [19]. Pin configuration consisted of parallel lateral pins in 12 patients, divergent lateral pins in 3, crossed pins in 4, and two lateral and one medial pin in 2 patients. The pin configuration was based on surgeon preference. We did not study the pin configuration in the remaining 469 children who did not develop pin tract infection. Preoperative antibiotics were not administered in 9 of 19 patients (47 %); data were missing in two patients. The average duration between arrival in the emergency department and time of surgery was 34.3 ± 76.5 h (range 1 h–14 days). Excluding one patient for whom an accurate time could not be assessed and three patients who underwent surgery due to loss of reduction 3, 5, and 14 days after initial closed reduction and cast application, the mean surgical delay was 9.3 ± 7.4 h (range 1–23 h).

There were 15 (3.1 %) superficial infections and 6 (1.2 %) deep infections (one osteomyelitis with septic arthritis, one osteomyelitis, one septic arthritis, and three deep soft tissue infections). Among the 15 patients with superficial infection, pin configuration involved parallel lateral pin placement in 7 patients, divergent lateral pins in 3, crossed pins in 3, and two parallel lateral and one medial pin in 2 patients. Of the patients diagnosed with deep infection, five had lateral parallel pins and one had crossed pins (Table 1). Patients presented with a variety of complaints, including continued or increasing pain or irritation (8 patients), discharge from the pin site (11 patients), fever (5 patients), constitutional symptoms of irritability, lethargy, loss of appetite or fatigue (3 patients), swelling (2 patients), loose or migrated pins (4 patients), and radiographic lucency on follow-up (1 patient).

**Table 1:** Demographics of patients with deep infection

Patient	Age (years)	Fracture side	Fracture type	Perioperative antibiotics	Surgical delay	Pin configuration	Complication	Presentation	Offending organism	Treatment	Follow-up (years)	DASH	PREE
1	5	Left	Type III	None	14 days	Parallel lateral	Deep soft tissue infection	Discharge, pain, fever	*Staphylococcus aureus*	Pin removal, I&D, IV and PO ATB	21	0	0
2	4.5	Right	Type III	Preoperative Ancef	3 h	Crossed	Deep soft tissue infection	Irritation beneath cast	*Staphylococcus aureus*	Pin removal, I&D, IV and PO ATB	19	–	–
3	8	Left	Type II	Postoperative Ancef	4 h	Parallel lateral	Septic bursitis	Fever, discharge, fatigue, loss of appetite	Unknown	Pin removal, I&D of sinus tract & bursa, IV and PO ATB	15	3	2
4	5	Right	Type II	Postoperative Keflex	20 h	Parallel lateral	Osteomyelitis, Septic arthritis	Mild pain, radiographic lucency	Unknown	I&D of distal humerus and elbow joint, IV ATB	17.5	0	0
5	6	Right	Type III	None	7 h	Parallel lateral	Septic arthritis	Discharge, swelling	*Pseudomonas aeruginosa*	I&D of sinus tract, IV ATB	16.5	0	0
6	5	Left	Type III	None	1 h	Parallel lateral	Osteomyelitis	Persistent discharge after removal of pins	*Pseudomonas aeruginosa*	I&D of sinus tract, IV and PO ATB	24	0	0

*I&D* irrigation and debridement, *ATB* antibiotics, *IV* intravenous, *PO* per os, *DASH* Disabilities of the Arm, Shoulder and Hand, *PREE* Patient-Rated Elbow Evaluation

All superficial infections were treated with pin removal after 3–4 postoperative weeks; a 7–10-day course of oral antibiotics, most commonly a second-generation cephalosporin; and local wound care. An above-elbow splint was applied in cases of early pin removal if fracture healing was not adequate. All superficial infections resolved without recurrence. There was no loss of fracture reduction following pin removal. For deep infections, all children underwent formal irrigation and debridement (including arthrotomy for those with septic arthritis) and a 6-week course of intravenous (2 patients) or a combination of intravenous and oral antibiotics (4 patients). Patient presentation, fracture type, pin configuration, and type of treatment for cases with deep infection are described in Table 1. At latest clinical follow-up, the infection had resolved in all patients. Patients had achieved full range of motion, and there was no clinical deformity. Follow-up radiographs showed complete healing of the fracture and no deformity.

An attempt was made to contact all patients for a telephonic interview at a mean 18 years (range 12–24 years) after surgery. Fifteen of the 21 patients (71 %) were interviewed. Ten of these patients had superficial pin tract infections and five had deep infections. DASH scores were excellent in all patients (mean 0.3 ± 0.5; range 0–3), as were PREE results (mean 0.2 ± 0.3; range 0–2). All patients were pleased with the cosmetic appearance of the arm with regard to carrying angle and scar site. All patients reported a full range of motion equal to that of the opposite extremity, no pain, and no need for analgesics. No patient reported any limitations with activities.

### Cadaveric study

Arthrograms confirmed that the medial pin was always extracapsular. However, because of the overlapping capsular anatomy on the lateral condyle, it was not always possible to determine capsular penetration by lateral pins on arthrograms.

**Fig. 4:**
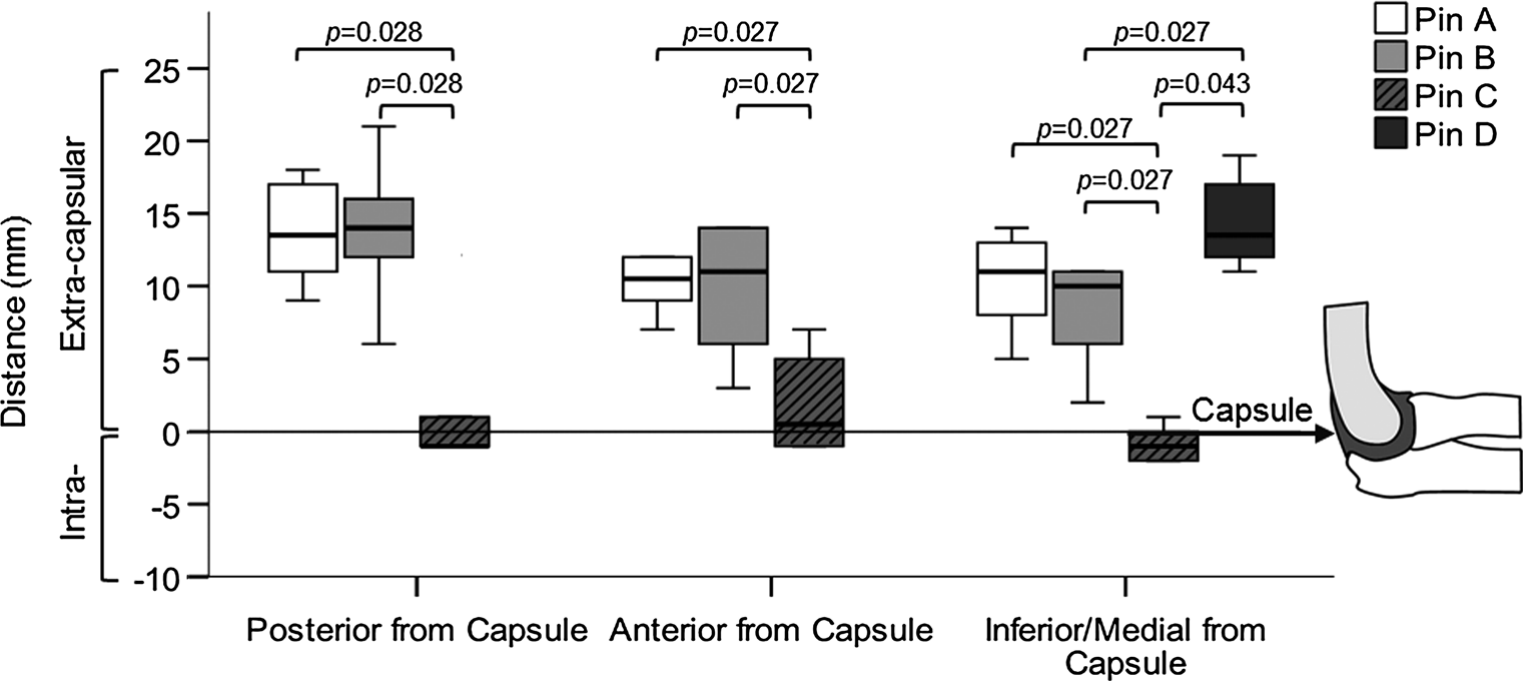
The four pin configurations (pin A = *lateral*, pin B = *lateral divergent*, pin C = *lateral parallel*, pin D = *medial crossed*) differed in their distance from the elbow capsule in the posterior, anterior, and inferior/medial directions. The ‘*zero*’ *line* represents capsular insertion. All pins except pin C were extracapsular in all specimens. Pin C was intracapsular in 4 of 6 specimens and on the capsule in 1 of 6 specimens. Statistically significant differences (*p* < 0.05) in distance between the pin configurations are indicated

After dissection, the median distance between pin A and the inferior joint capsule was 11.0 mm, while the median distance between pin B and the inferior joint capsule was 10.0 mm. The median distance between pin C and the inferior joint capsule was −1.0 mm, with the negative value indicating intracapsular placement. For parallel pin configuration, pin C was intracapsular in four of the six specimens, extracapsular in one, and on the capsule in another. The risk of intra-articular pin placement in parallel pins was significantly greater (*p* < 0.05) than in the other two configurations (Fig. 4). The median distance of the lateral pins (pins A, B and C) from the anterior, posterior, and inferior joint capsule is shown in Table 2. The median distance between pin D and the medial joint capsule was 13.5 mm.

**Table 2:** Pin distance from the elbow capsule

Pin	Pin configuration	Posterior from capsule	Anterior from capsule	Inferior/medial from capsule
		Median (IQR)	Median (IQR)	Median (IQR)
A	Lateral (reference)	13.5 (10.5–17.3)	10.5 (8.5–13.3)	11.0 (7.3–13.3)
B	Lateral divergent	14.0 (10.5–17.3)	11.0 (5.3–14.0)	10.0 (5.0–11.0)
C	Lateral parallel	−1.0 (−1.0 to 3.8)*^†^	0.5 (−1.0 to 5.5)*^†^	−1.0 (−2.0 to 0.3)*^†‡^
D	Medial (crossed pin)	–	–	13.5 (11.8–17.5)^†^

*IQR* interquartile range; negative values indicate intracapsular penetration

Significant difference (*p* < 0.05) compared to * pin A, ^†^ pin B, and ^‡^ pin D based on two-tailed Wilcoxon signed-rank test

## Discussion

A few studies have reported the incidence and management of pin tract infection following pinning of supracondylar humerus fractures in children. Pirone et al. [2] reported two cases of superficial pin tract infection in a series of 96 patients treated with closed reduction and pinning. Boyd et al. [20] described two patients in their series of 99 patients, one with an excellent outcome following irrigation and debridement of pin tract infection, and another who developed osteomyelitis with septic arthritis. In the latter case, the outcome was unsatisfactory secondary to loss of motion. Iobst et al. [14] reported no infections in 304 cases treated at a single institution using the semi-sterile technique. The authors reviewed the literature and reported an overall pin tract infection rate of 2.3 % (45/1922). Skaags et al. [4] reported a 2.1 % rate of infection in a series of 189 supracondylar type II fractures. Mehlman et al. [15] reported five pin tract infections, with no significant difference in fractures treated early (less than 8 h) versus those treated late. More recently, Bashyal et al. [6] described the results of supracondylar fracture pinning at a single institution, noting a total infection rate of 1 % (6 of 622) and a deep infection rate of 0.2 %. They found no advantage in full preparation and draping or preoperative antibiotics. Sharma et al. [13] noted higher pin-related complications when the pins were left outside the skin for a longer duration and when pins did not traverse both cortices.

Infection after percutaneous pinning of supracondylar fractures is a not an uncommon event, as evidenced by our study. Our overall incidence of 4.3 % is slightly higher compared to other reports in the literature, which may have been due to bundling of pin tract irritation, hypergranulation tissue, and pin loosening/migration in the pin tract infection group. Most of these infections were superficial and were treated with oral antibiotics and wound care. However, the procedure is not completely benign, as evident in six of our patients who developed deep infections requiring surgical debridement and intravenous antibiotics. The recognition and prompt treatment of pin tract infection is of utmost importance. Despite the infections, patients reported excellent functional results at skeletal maturity at a mean 18 years after the index procedure. Five of six (83 %) patients with deep infection had parallel lateral pins. However, two deep infections presented late, and one was lost to follow-up. Also, we did not study pin configuration in the remaining 469 children who did not develop infection, and thus cannot establish a causal relationship between infection and parallel pinning.

The optimal pin configuration is a subject of considerable debate in relation to ulnar nerve injury and biomechanical principles [3]. Randomized clinical trials have shown that lateral-entry pin fixation and crossed-pin configuration are equally effective in the treatment of displaced supracondylar fractures [21, 22]. However, the risk of iatrogenic ulnar nerve injury following percutaneous fixation using a crossed-pin technique has been well documented [8–10]. Many authors have concluded that fixation with lateral pins is a safer and more effective method for displaced supracondylar fractures in children [11, 23–25]. Studies have also demonstrated that the site of pin insertion is less important for fracture stability than the site at which the pins cross the fracture [5, 26, 27]. Kallio et al. [28], who focused on optimal lateral pin placement technique, recommended divergent pin placement for maximum stability and avoidance of joint penetration. In a biomechanical comparison of all three configurations, Lee et al. [29] reported that divergent lateral pin configuration provided greater stability than parallel pin configuration, and demonstrated similar stability compared with crossed-pin configuration. This was confirmed by another biomechanical study showing that the best torsional, valgus, and extension resistance are associated with the most divergent configuration of the lateral pins, in which the diverging pin crosses the fracture site at the medial edge of the coronoid fossa [30]. Based on the results of our cadaveric study, parallel lateral pin configuration may increase the risk of intracapsular pin placement compared to divergent lateral pin configuration. Thus, of the three configurations, divergent lateral pin placement is most desirable, since it has less potential for nerve injury, ensures optimal fracture stability, and in addition reduces the risk of intracapsular pin placement.

This study does have certain limitations. Since it involved procedures performed over the period 1983–2002, the standard of treatment and approach to treating these fractures, including the use of preoperative antibiotics, varied among studied cases. Due to the retrospective study method employed, we had to rely on medical records, which were not always complete. Many patients who were contacted by phone had moved, and radiological or clinical follow-up was not feasible. Because of the difficulty in procuring pediatric cadaveric elbows, the cadaveric study was performed on adult elbows. Thus, absolute measurements would differ in pediatric patients. Still, it is reasonable to accept that divergent lateral pinning is safer than parallel lateral pinning for avoiding intra-articular placement of pins. The association between intracapsular pin placement and deep infection cannot be established from the current study. Similarly, the association of other risk factors such as preoperative antibiotics, time to surgery, and host-related factors with pin tract infection could not be established from our results.

In summary, most infections that occur after pinning of supracondylar humerus fractures are superficial and can be managed with oral antibiotics and local wound care. In rare cases of osteomyelitis or septic arthritis, satisfactory long-term functional outcomes can be expected when treated aggressively with surgical debridement and intravenous antibiotics. The parallel lateral pin configuration may increase the risk of capsular penetration. Intracapsular pin placement can be avoided by divergent lateral pin or crossed-pin configuration.
